# Intestinal T-cell Responses in Celiac Disease – Impact of Celiac Disease Associated Bacteria

**DOI:** 10.1371/journal.pone.0053414

**Published:** 2013-01-09

**Authors:** Veronika Sjöberg, Olof Sandström, Maria Hedberg, Sten Hammarström, Olle Hernell, Marie-Louise Hammarström

**Affiliations:** 1 Department of Clinical Microbiology, Immunology, Umeå University, Umeå, Sweden; 2 Department of Clinical Sciences, Pediatrics, Umeå University, Umeå, Sweden; Tulane University, United States of America

## Abstract

A hallmark of active celiac disease (CD), an inflammatory small-bowel enteropathy caused by permanent intolerance to gluten, is cytokine production by intestinal T lymphocytes. Prerequisites for contracting CD are that the individual carries the MHC class II alleles HLA-DQ2 and/or HLA-DQ8 and is exposed to gluten in the diet. Dysbiosis in the resident microbiota has been suggested to be another risk factor for CD. In fact, rod shaped bacteria adhering to the small intestinal mucosa were frequently seen in patients with CD during the “Swedish CD epidemic” and bacterial candidates could later be isolated from patients born during the epidemic suggesting long-lasting changes in the gut microbiota. Interleukin-17A (IL-17A) plays a role in both inflammation and anti-bacterial responses. In active CD IL-17A was produced by both CD8^+^ T cells (Tc17) and CD4^+^ T cells (Th17), with intraepithelial Tc17 cells being the dominant producers. Gluten peptides as well as CD associated bacteria induced IL-17A responses in *ex vivo* challenged biopsies from patients with inactive CD. The IL-17A response was suppressed in patients born during the epidemic when a mixture of CD associated bacteria was added to gluten, while the reverse was the case in patients born after the epidemic. Under these conditions Th17 cells were the dominant producers. Thus Tc17 and Th17 responses to gluten and bacteria seem to pave the way for the chronic disease with interferon-γ-production by intraepithelial Tc1 cells and lamina propria Th1 cells. The CD associated bacteria and the dysbiosis they might cause in the resident microbiota may be a risk factor for CD either by directly influencing the immune responses in the mucosa or by enhancing inflammatory responses to gluten.

## Introduction

Celiac disease (CD) is a chronic inflammatory disease, caused by failure to establish and/or maintain tolerance to the food antigen gluten in wheat and related prolamines in barley and rye [1], [2]. The disease affects genetically susceptible individuals carrying the MHC class II alleles HLA-DQ2 and/or HLA-DQ8 [1]. In CD patients, intake of gluten causes an inflammatory lesion in the small intestine characterized by villous atrophy and crypt hyperplasia in combination with increased frequency of T cells within both the epithelium and the lamina propria (LP) [1]–[3]. Other features of active CD are production of IgA antibodies to gliadin, a protein fraction of gluten (IgA-AGA) and the autoantigen tissue transglutaminase (IgA-tTG), up-regulation of both the pro-inflammatory cytokine interferon (IFN)-γ and the down-regulatory cytokine interleukin (IL)-10 [1], [3], [4]. Clinical and histological improvement as well as normalisation of IgA-AGA and IgA-tTG serum titers and cessation of cytokine over-production are seen upon withdrawal of gluten from the diet, which presently is the only treatment of CD [2], [5].

T cells, both within the epithelium and the LP, are key players in the pathogenesis of CD [4], [6]. CD4^+^- and CD8^+^ gluten specific T cell clones have been isolated from the small intestinal mucosa of CD patients [7], [8]. Intraepithelial T lymphocytes, particularly CD8^+^ cells, are the major contributors of IFN-γ and IL-10 in biopsies collected from CD patients with active disease at diagnosis while the relative contribution from T cells in the LP increases in active CD caused by challenge with gluten after a symptom-free period on gluten-free diet [4], [9]. Furthermore, children with CD have impaired capacity for extrathymic T cell receptor (TCR) rearrangement suggesting that the failure to establish tolerance to gluten is at least partly due to reduced capacity to generate T cells locally in the small intestinal mucosa [10].

Over the years 1985–1996 the incidence of CD showed a four-fold increase followed by a rapid decrease in Swedish children below 2 years of age [11]. This period is referred to as the “Swedish CD epidemic”. Although part of the epidemic can be explained by altered infant feeding mode resulting from changed national recommendations [12], [13] it has features of an infectious disease. In fact, we have found that the small intestinal microbiota of children with CD born during the epidemic was different from that of children with CD born after the epidemic and controls, with a significant enrichment of rod-shaped bacteria of the *Lachnoanaerobaculum*, *Prevotella,* and *Actinomyces* genera [14]–[16]. Possibly, any or all of these bacteria may contribute to the aetiology and/or pathogenesis of childhood CD, by causing an undesired dysbiosis during establishment of the gut microbiota.

IL-17A is involved in antibacterial defense by recruiting immune cells to sites of infection but also acts as a pro-inflammatory cytokine in autoimmune and inflammatory diseases [17], [18]. Several studies suggest a role for IL-17A also in CD. Increased levels of IL-17A were reported in the small intestinal mucosa of patients with active disease and after *ex vivo* challenge with gliadin [19]. However, studies on IL-17A producing CD4^+^ T cells, i.e. Th17 cells, are contradictory. Two groups reported presence of gliadin specific Th17 cells in the small intestinal mucosa of CD patients [20], [21] while one group reported that challenge with either gliadin or polyclonal T cell activators does not induce IL-17A production in established gliadin specific CD4^+^ T cell lines [22]. Furthermore, La Scaleia et al [23] recently reported that polyclonally stimulated CD4^+^ T cells of CD patients do not produce IL-17A. Possibly differences in the composition of the resident microbiota contribute to the contradictory results concerning Th17 responses in CD.

We hypothesize that dysregulation of pro-inflammatory cytokine producing lymphocytes and down-regulatory regulatory T cells (Tregs) might contribute to failure of establishing and/or maintaining oral tolerance to gluten resulting in development of CD. Moreover, we hypothesize that the CD associated bacteria and/or the dysbiosis in the resident microbiota that they may cause, is a risk factor for CD either by directly influencing the immune responses in the mucosa or by enhancing inflammatory responses to gluten. In this study we determined the cellular source of IL-17A in untreated CD, explored whether the balance between IL-17A production and Treg activity has a role in the pathogenesis of CD and investigated whether the CD associated bacteria induce IL-17A production and/or influence the IL-17A response to gluten. The anti-bacterial immune response was assessed by *ex vivo* challenge of biopsies from treated CD patients with CD associated bacteria alone and in combination with gluten.

## Materials and Methods

### Ethics Statement

All biopsies were taken as part of clinical examination with the suspicion of CD or with the intention to exclude the diagnosis. Written informed consent was obtained from the parents of participating children with permission to use biological material in the present study. The local Research Ethics Committee of the Faculty of Medicine, Umeå University approved the study, including the consent form.

### Patients and Biopsy Sampling

Small intestinal biopsies were collected sequentially from children admitted to the Department of Pediatrics, Umeå University Hospital on suspicion of CD, or for evaluation of asymptomatic growth failure, short stature or abdominal pain. Biopsies were taken from distal duodenum/proximal jejunum, using a Watson pediatric capsule or by endoscopic procedure and immediately placed in ice-chilled HEPES-buffered RPMI 1640. Part of a biopsy was used for routine pathology examination and the rest used for *ex vivo* challenge, RNA extraction, immunohistochemistry, or isolation of T cell subsets of intraepithelial lymphocytes (IELs) and lamina propria lymphocytes (LPLs). Patients belonged to one of three diagnostic groups; Untreated CD: Fourteen boys and 28 girls [7.5 (4–12) years (median and range)] showing serum IgA-tTG levels >5 U/ml and small intestinal mucosa morphology with histological changes classified as Marsh score IIIa-c [24]. All responded positively to a gluten-free diet. Treated CD: Eleven boys and 18 girls [7.5 (5.3–14.5) years] with an IgA-tTG level ≤4 U/ml and Marsh score 0, who had been on a gluten-free diet for ≥5 months. Clinical controls: 14 boys and 15 girls [5.5 (2–11.3) years] with no known food intolerance and with serum IgA-tTG levels ≤4 and Marsh score 0.

### CD Associated Bacteria

Eight bacterial isolates from proximal jejunum of two CD patients (*Prevotella* isolates CD3∶27, CD3∶28, CD3∶32, CD3∶33, CD3∶34, *Lachnoanaerobaculum umeaense* isolate CD3∶22, and *Actinomyces graevenitzii* isolate CD4:Bx9) and one control (*Prevotella* isolate CD116∶18) [15], [16] were cultivated under anaerobic conditions (10% H_2_ and 5% CO_2_ in N_2_) on blood agar plates (Colombia Blood Agar Base [Acumedia] supplemented with 5% defibrinated horse blood). Colonies were picked and washed in phosphate buffered saline (PBS; pH 7.4) and the density was adjusted to 4 on the McFarland optical scale, representing 1.2×10^9^ bacteria/ml. Each bacterial isolate was suspended in 1 ml of challenge medium (one part RPMI 1640 containing 0.4% human serum albumin and one part Medium199 containing 15% human normal AB^+^ serum, 2 mM Na-pyruvate and a supplement of non-essential amino acids), and thereafter 100 µl of each bacterial isolate suspension was added together.

### *Ex vivo* Challenge of Biopsies with Gluten Digest and CD Associated Bacteria

Freshly collected small intestinal biopsies from symptom-free CD patients on gluten-free diet (Treated CD) were divided into five or six small pieces. One piece was directly frozen for RNA extraction and stored at −80°C to be used for control of the *in vivo* immune status. The other four pieces were incubated in parallel with either challenge medium alone (medium control) or challenge medium supplemented with trypsin treated gluten (1 mg/ml) [25] and/or 1.1×10^8^ small intestinal CD associated bacteria mix/well in a total volume of 1.1 ml in 5% CO_2_ at 37°C for 24 h. These anaerobic bacteria do not grow under these conditions. After incubation the biopsy pieces were frozen and stored at −80°C until RNA extraction. In two cases IEL and LPL T cell subsets were isolated from one biopsy piece, challenged with the combination of gluten digest and the mixture of CD associated bacteria.

### Cell Isolation Procedures

IELs and LPLs were isolated separately from small intestinal biopsies, fresh or challenged *ex vivo* with a combination of gluten digest and the mixture of CD associated bacteria. IELs and LPLs were subdivided into TCR-γδ^+^ cells (γδ^+^IELs, γδ^+^LPLs), CD4^+^TCR-γδ^–^ cells (CD4^+^IELs, CD4^+^LPLs), CD8^+^TCR-γδ^−^ CD4^−^ cells (CD8^+^IELs, CD8^+^LPLs) by sequential, positive selection to anti-TCR-γδ, anti-CD4, and anti-CD8 charged magnetic beads as described [9]. This method yields more than 98% pure preparations of the marker positive cells [26].

### RNA Extraction

Total RNA was extracted from biopsies and T cell subsets by the acid guanidinium thiocyanate/phenol/chloroform method or by using the RNeasy Mini Kit (Qiagen, Sollentuna, Sweden) and dissolved in RNase-free water containing rRNasin ribonuclease inhibitor (Promega, Madison, WI), as described [27].

### Real-time Quantitative Reverse Transcriptase-polymerase Chain Reaction (qRT-PCR)

Quantification of IL-17A, IFN-γ, IL-10, transforming growth factor-β1 (TGF-β1, and Foxp3 mRNAs was performed using real-time qRT-PCR assays constructed in the laboratory based on the EZ-technology, with primers placed in different exons, a reporter dye marked probe placed over the exon boundary in the amplicon and an RNA copy standard. The sequences in the Foxp3 assay were from Morgan et al. [28]. For details of the IFN-γ, IL-10, and TGF-β1 assays see Forsberg et al. [4] and for details of the IL-17A assay see West et al. [29]. Samples were analyzed in triplicate and expressed as mRNA copies/µl. The concentration of 18S rRNA was determined in each sample using real-time qRT-PCR (Applied Biosystems, Foster City, CA) and expressed as arbitrary units (U) from a standard curve of serial dilutions of a preparation of total RNA from human peripheral blood mononuclear cells. One U was defined as the amount of 18S rRNA in 10 pg RNA and corresponds to approximately 100 lymphocytes [30]. In biopsies the 18S rRNA from lymphocytes is diluted in the excess of 18S rRNA from other cell types in the tissue, e.g. epithelial- and stromal cells, yielding at least 10–100 fold lower mRNA levels than in purified T lymphocyte subsets. mRNA concentrations were normalized to the18S rRNA concentration in the sample and results are expressed as mRNA copies/18S rRNA U. All samples included in the study contained >16 U 18S rRNA per reaction mixture.

### Immunomorphometry

Fresh biopsy pieces were snap frozen in embedding medium (Tissue-tek O.C.T compound, Sakura Finetek, Zoeterwoude, Netherlands) and stored at −80°C. Cryosections were stained as previously described with the modification that 1% Triton-X100 was included in the blocking buffer [10]. The antibodies used were mouse anti-human IL-17A monoclonal antibody (mAb) (clone BL-168, IgG1; BioLegend, San Diego, CA) and mouse anti-human Foxp3 mAb (clone236A/E7, IgG1; Abcam, Cambridge, MA). Concentration-matched irrelevant mouse mAb of IgG1 class (X0931, DakoCytomation, Glosterup, Denmark), served as negative control, and mouse anti-human CD45 mAbs (clones 2B11 and PD7/26, IgG1) served as positive control. Morphometry analysis for frequencies of IL-17A and Foxp3 positive IELs and LPLs were performed by inspection of the epithelium and the LP separately for positively stained IELs and LPLs, respectively. The entire area of the epithelium and the LP in each tissue section was measured using an integrating, cooled color 3CCD camera (Color Chilled 3 CCD Hamamatsu Camera C5810; Hamamatsu Photonics, Hamamatsu City, Japan) on a standard light microscope combined with an interactive computer image analysis system (LeicaQWin; Leica Imaging Systems, Cambridge, UK). The results are expressed as numbers of positively stained cells per mm^2^.

### Two-color Immunofluorescence Staining

Cryostat sections (10 µm thick) of fresh frozen pieces of biopsies were air-dried, fixed in 4% paraformaldehyde for 15 min at room temperature, rinsed in cold 0.02 M PBS (pH 7.4) and blocked with 0.1 M glycine in PBS followed by PBS containing 0.2% bovine serum albumin/0.05% saponin/1% Triton-X100 and finally incubated with 2.5% normal horse serum (Vector Laboratories, Burlingame, CA). Thereafter the sections were incubated with mouse anti-human IL-17A mAb for 15 min at 37°C followed by 45 min at 4°C, washed in PBS and incubated with FITC-conjugated affinity-purified F(ab)_2_ fragments of goat anti-mouse IgG+IgM (H+L) (Jackson Immunoresearch Laboratories, West Grove, PA) for 45 min. The sections were then washed in PBS and blocked with streptavidin for 15 min, rinsed and blocked with biotin for 15 min (Vector Laboratories). Finally the sections were incubated for 1 h at room temperature with biotin conjugated mouse anti-human CD8 mAb (clone RFT-8, IgG, Abcam), washed with PBS and incubated with RPE-conjugated streptavidin (DakoCytomation) for 45 min. After rinsing with PBS the sections were mounted in Immunoconcept mounting media (Immunoconcept, Bonn, Germany) and inspected under a fluorescence microscope (Leica DMBR, Wetzlar, Germany).

### Statistics

Statistical analyses were performed using the Prism 5 computer program (GraphPad Software, San Diego, CA). Statistical analysis of differences in frequencies of cells stained by immunohistochemistry and in mRNA expression levels between two groups were performed using two-sided Mann Whitney U-test. Comparisons of mRNA expression levels between three groups were performed using Kruskal-Walli’s one-way analysis of variance (ANOVA) test with the Dunn’s Multiple Comparison post-test. Comparisons of relative contribution of mRNA by the three IEL subsets were performed using one-way ANOVA with Bonferroni’s Multiple Comparison post-test. Analyses of correlation between mRNA expression levels of different cytokines were performed using two-tailed Spearman rank correlation test. A *P*-value <0.05 was regarded statistically significant. Descriptive values for frequencies of cells and mRNA expression levels are given as median, IQR from the 25th to the 75th percentile and range and for relative contribution of mRNA by IEL subsets as mean ±1 SD.

## Results

### mRNA Levels of IL-17A, IFN-γ, IL-10, and the Treg Marker Foxp3 all Follow the Disease Activity in the Small Intestinal Mucosa of Children with CD

**Figure 1:**
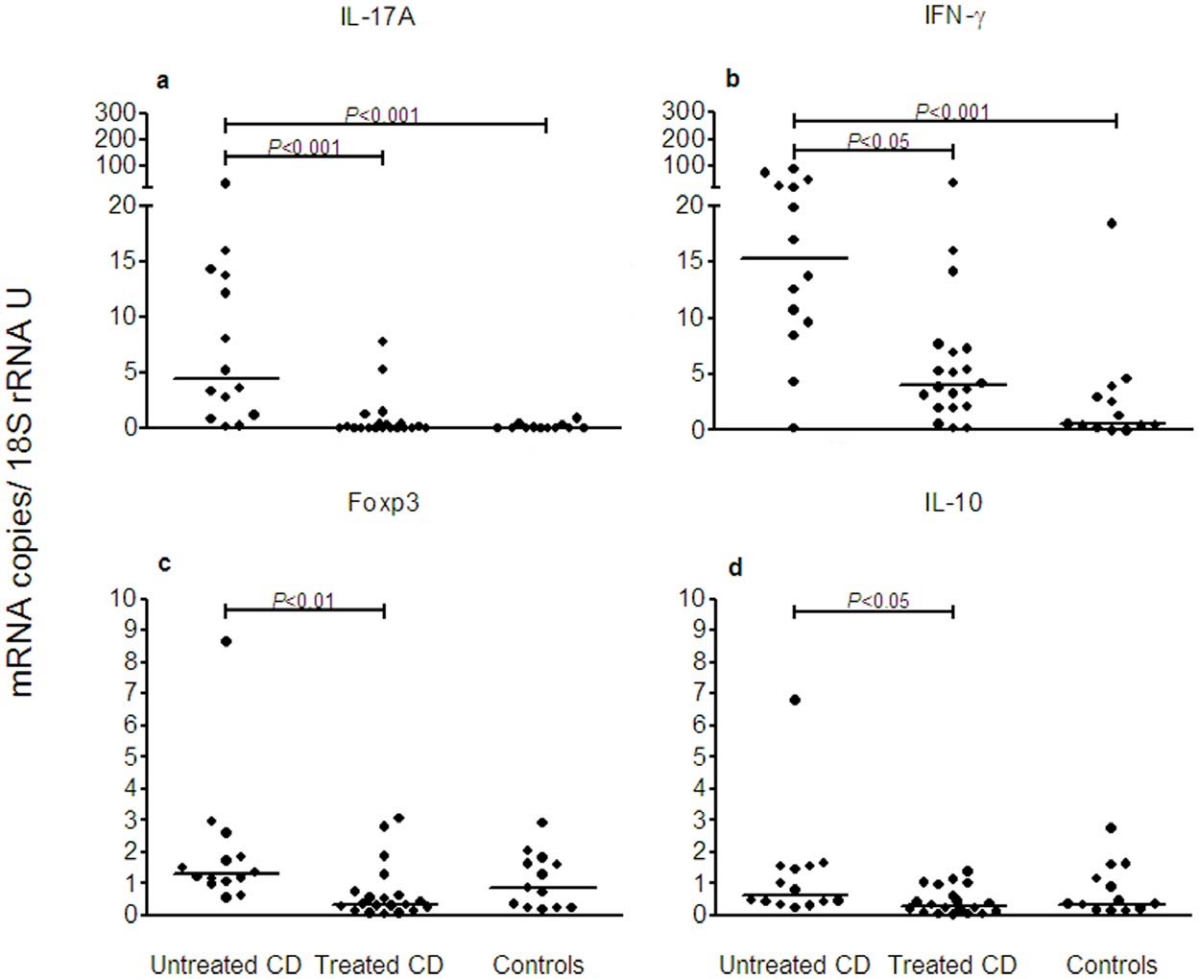
IL-17A mRNA levels are elevated in patients with active CD. Expression levels of mRNAs for IL-17A (**a**), IFN-γ (**b**), Foxp3 (**c**) and IL-10 (**d**) were determined in freshly taken biopsies from the proximal small intestine of pediatric CD patients with untreated, active disease (Untreated CD), CD patients on gluten-free diet with inactive disease (Treated CD) and clinical controls (Controls) using qRT-PCR. Amounts of cytokine and Foxp3 mRNAs were normalized to the 18S rRNA content in each sample. Dots depict expression levels in individual samples. Horizontal bars indicate median values. *P*-values of statistically significant differences are depicted.

Small intestinal biopsies of children on a gluten-containing diet with active disease and later confirmed to have CD (Untreated CD), children with CD on a gluten-free diet and with inactive disease (Treated CD), and pediatric, clinical controls with no known food intolerance (Controls) were analyzed for mRNA levels of the cytokines IL-17A, IFN-γ, IL-10, and TGF-β1, and the Treg marker Foxp3. Levels of IL-17A and IFN-γ were significantly higher in biopsies from patients with Untreated CD compared to both Treated CD and Controls (**Figure** 1a, b). A difference in expression pattern of the two cytokines was however seen. In Treated CD, the median level of IL-17A was the same as in Controls (**Figure** 1a), while the median level of IFN-γ tended to be higher than in Controls (ratio between median for Treated CD and Controls: 4.4; **Figure** 1b). Foxp3, IL-10, and TGF-β1 all showed a similar pattern with significantly higher levels in Untreated CD compared to Treated CD, but not to Controls (**Figure** 1c, d and data not shown).

Pair-wise correlation analyses between the mRNA levels of the cytokines and Foxp3 in biopsies of the three study-groups revealed significant correlation between IL-17A and both IFN-γ and Foxp3 in Untreated CD and between Foxp3 and TGF-β1 in Treated CD (Table 1). In Controls there was no correlation between any of the cytokine mRNAs or between these and Foxp3 mRNA (data not shown).

**Table 1:** IL-17A, IFN-γ and Foxp3 mRNA levels correlate in small intestinal mucosa of patients with untreated celiac disease (Untreated CD) but not in symptom-free celiac disease patients on gluten-free diet (Treated CD).

	Untreated CD		Treated CD	
mRNA species compared:	r	*P*-value	r	*P*-value
IL-17A versus IFN-γ^1^	0.53^2^	**0.05** 2	0.01	NS^3^
IL-17A versus Foxp3	0.61	**0.02**	0.10	NS
IL-17A versus IL-10	0.42	NS	0.20	NS
IL-17A versus TGF-β1	0.12	NS	0.18	NS
Foxp3 versus IFN-γ	0.02	NS	0.38	NS
Foxp3 versus IL-10	0.27	NS	0.17	NS
Foxp3 versus TGF-β1	0.04	NS	0.52	**0.02**

1 mRNA expression levels were compared in each individual sample.

2 r- and *P*-values obtained by correlation analysis between indicated mRNA species as determined by two-tailed Spearman rank correlation test.

3 NS = not significant, *P*-value >0.05.

### Frequencies of IL-17A Producing Lymphocytes are Increased in Untreated CD

**Figure 2:**
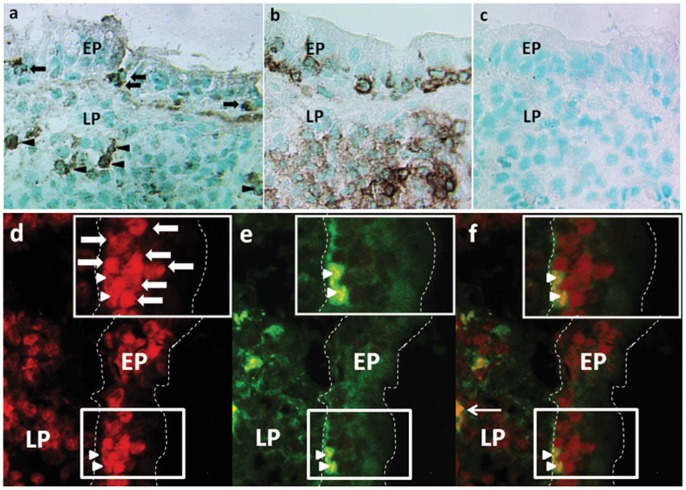
Tc17 cells are present in the small intestinal mucosa of patients with active CD. Immunoperoxidase (**a**–**c**) and immunofluorescence (**d**–**f**) staining of the small intestinal mucosa of patients with untreated CD. (**a**–**c**) sequential sections of intestinal mucosa of one CD patient with active disease stained with anti-IL-17A mAb (**a**) showing cells with cytoplasmic staining for IL-17A both within the epithelium (black arrows) and in the LP (black arrowheads), suggesting IL-17A secretion at both locations; (**b**), positive control, i.e. section stained with anti-CD45 mAbs identifying all leukocytes; (**c**), negative control, i.e. section incubated with concentration and isotype matched irrelevant mAb. (**d**–**f**) a cryosection of intestinal mucosa of one CD patient with active disease stained in two-color immunofluorescence. (**d**), CD8^+^ cells stained in red. (**e**), IL-17A^+^ cells stained in green. (**f**), overlay of (**d**) and (**e**) showing yellow IL-17A^+^CD8^+^ cells. The box in (**d**) encloses a cluster of 9 CD8^+^ IELs, two of them are also positive for IL-17A (**e**, **f**). Thick white arrows depict CD8 single positive IELs. White arrowheads depict CD8/IL-17A double positive IELs. Thin white arrow depicts a CD8/IL-17A double positive LPL. Original magnification, X 220 (**a**–**c**) and X 400 (**d**–**f**). EP indicates epithelium and LP lamina propria. Dotted white lines in d–f indicate the basal lamina and the apical surface of the epithelium. Smaller boxes in (**d**–**f**) indicate the area enlarged in the upper right corners.

Immunohistochemistry was used to verify IL-17A and Foxp3 expression at the protein level and to assess the distribution of lymphocytes expressing these proteins. IL-17A^+^ lymphocytes were detected both within the epithelium and in the LP (Figure 2a) constituting approximately 15 and 30% of the IELs and LPLs (CD45^+^ cells), respectively (Figure 2a, b). IL-17A^+^ IELs were found both as scattered single cells and in small clusters in the villous/luminal area of the epithelium, but were rare in the crypt epithelium. IL-17A^+^ LPLs were evenly distributed within the LP. Both IELs and LPLs showed two types of IL-17A staining pattern: evenly distributed cytoplasmic staining resulting in a positively stained ring around the nucleus, and cytoplasmic dots suggesting ongoing secretion. Several of the IL-17A^+^ IELs and LPLs were CD8^+^ cells as determined by two-color immunofluorescence staining of the small intestinal mucosa of patients with active CD (**Figure** 2d–f).

In agreement with the IL-17A mRNA analyses, the frequency of IL-17A^+^ cells was significantly increased in patients with Untreated CD compared to Controls. This increase was seen both within the epithelium and in the LP (Table 2) but was most pronounced within the epithelium with a 6.7-fold higher median frequency than in Controls. Comparison of the average frequencies of IL-17A^+^ cells in IELs and LPLs of patients with Untreated CD showed a 9.9-times higher frequency in IELs (Table 2).

**Table 2:** Frequencies of IL-17A^+^ lymphocytes are higher in small intestinal biopsies of patients with untreated celiac disease (Untreated CD) compared to controls both within the epithelium and in the lamina propria.

	Untreated CD			Controls			
Cell type^1^	Median	IQR	n	Median	IQR	n	*P*-value
**IL-17A^+^ IELs**	57.3^2^	51.1–64.8	6	8.6	3.0–11.6	6	0.002
**Foxp3^+^ IELs**	12.7	11.3–22.2	4	11.6	6.2–23.1	4	NS^3^
							
**IL-17A^+^ LPLs**	5.8	5.2–19.2	8	3.9	2.9–4.7	6	0.02
**Foxp3^+^ LPLs**	2.9	1.4–6.6	8	2.3	1.8–10.9	6	NS

1 Intraepithelial lymphocytes (IELs) and lamina propria lymphocytes (LPLs) positively stained for IL-17A (IL-17A^+^) and Foxp3 (Foxp3^+^) by indirect immunohistochemistry.

2 Number of positively stained cells per mm^2^.

3 NS = not significant, *P*-value >0.05.

Foxp3^+^ cells were detected both within the epithelium and in the LP in biopsies of Untreated CD and Controls (data not shown). There was no significant difference in frequencies of Foxp3^+^ cells between the two groups at either location (Table 2).

### IL-17A is Expressed at High Levels Both in CD8^+^- and CD4^+^ IELs in Untreated CD

**Figure 3:**
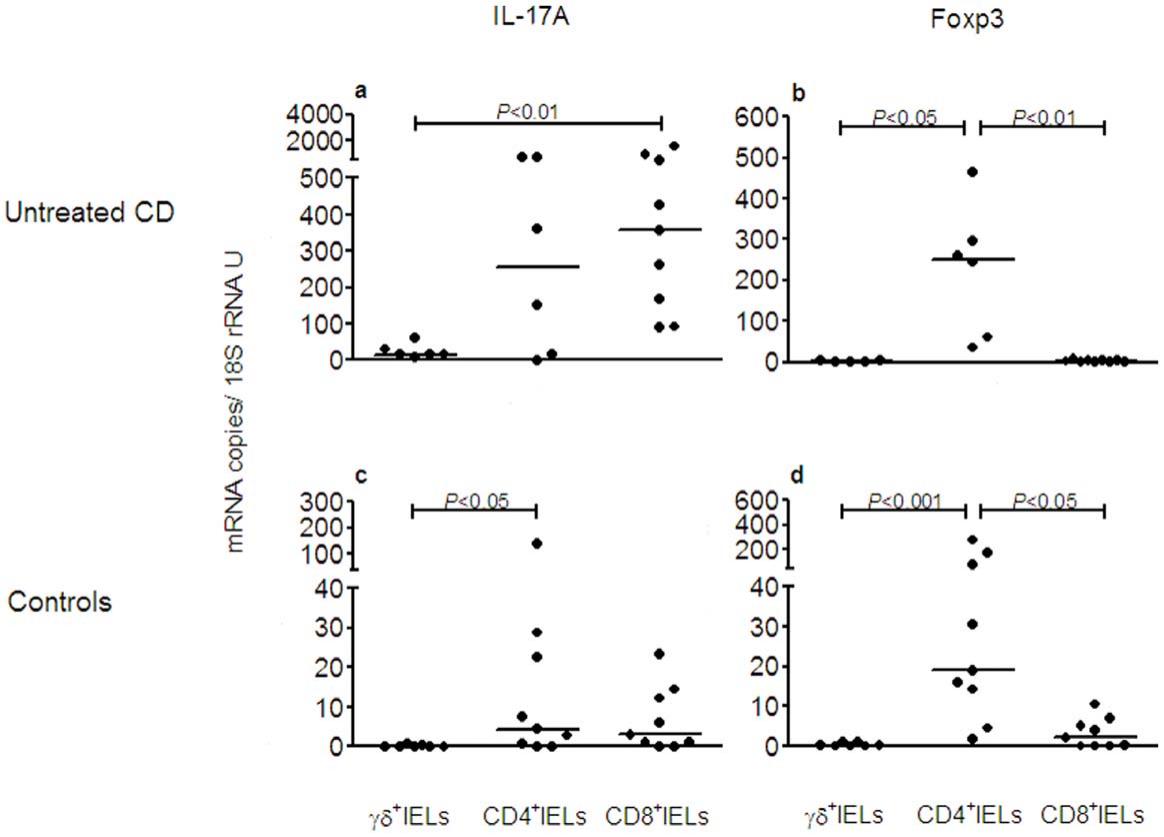
High levels of IL-17A mRNA in intraepithelial Tc17 and Th17 cells in active CD. Expression levels of IL-17A (**a**, **c**) and Foxp3 (**b**, **d**) mRNAs were determined in TCR-γδ^+^ IELs (γδ^+^IELs), CD4^+^ TCR-γδ^−^ IELs (CD4^+^IELs), and CD8^+^ CD4^−^ TCR-γδ^−^ IELs (CD8^+^IELs) retrieved from IELs isolated from intestinal biopsies of patients with untreated CD (Untreated CD) and controls (Controls). Dots depict expression levels for the indicated mRNA species in individual samples. Horizontal bars indicate median values. *P*-values of statistically significant differences are depicted.

IL-17A and Foxp3 mRNA expression levels were determined in γδ^+^IELs, CD4^+^IELs, and CD8^+^IELs obtained by cell-fractionation of lymphocytes from intestinal biopsies of patients with Untreated CD and Controls. In Controls, IL-17A mRNA was expressed at low levels in both CD4^+^- and CD8^+^IELs (median 4.5 and 3.1 mRNA copies/18S rRNA U, respectively; Figure 3c) while Foxp3 mRNA was expressed preferentially in CD4^+^IELs and at on average 4 to 6-fold higher levels than IL-17A (median 18.9 mRNA copies/18S rRNA U; Figure 3d).

In Untreated CD both CD4^+^- and CD8^+^IELs showed markedly higher average expression levels of IL-17A mRNA than the corresponding T cell subtype of Controls (average 57-fold and 115-fold difference in CD4^+^- and CD8^+^ IELs, respectively) and for CD8^+^IELs the difference reached statistical significance (*P*<0.001; Figure 3a and c). Although, the IL-17A mRNA level in γδ^+^IELs was low in Untreated CD it was still significantly higher than in Controls (*P*<0.01; Figure 3a and c).

**Figure 4:**
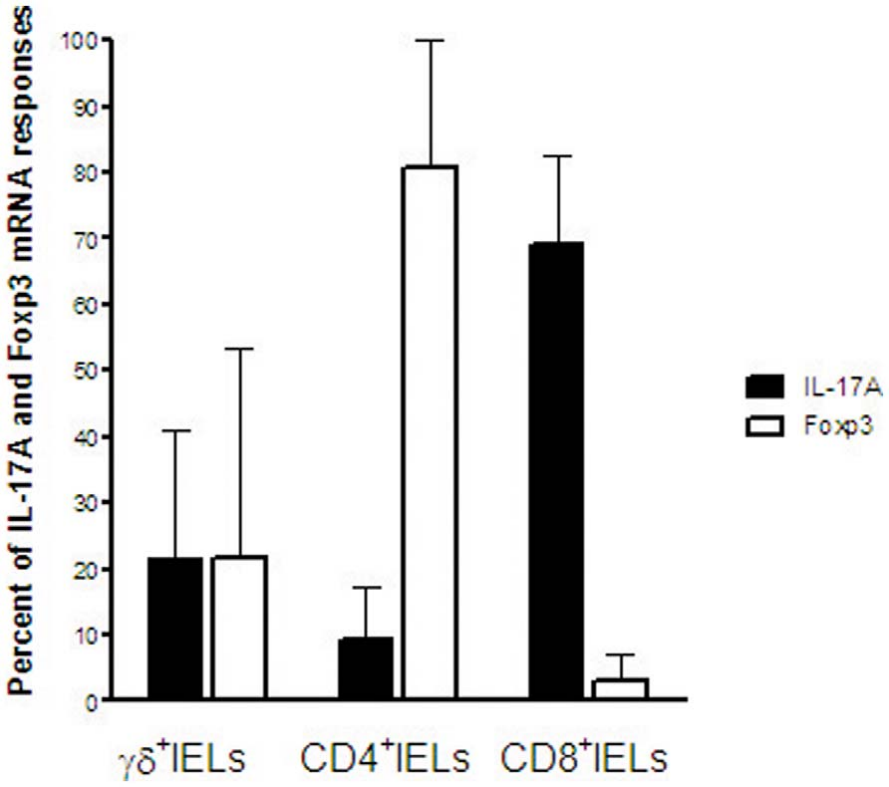
Tc17 cells contribute most of the IL-17A mRNA in the intestinal epithelium in active CD. TCR-γδ^+^IELs (γδ^+^IELs), CD4^+^IELs and CD8^+^IELs were sequentially retrieved from small intestinal biopsies of 4 patients with untreated CD and analyzed for IL-17A mRNA and Foxp3 mRNA. Results are expressed as relative contribution in percent to the total amount of mRNA of the indicated species in the γδ^+^IEL, CD4^+^IEL and CD8^+^IEL subsets of each individual. Bars indicate mean and whiskers 1 SD. Filled bars, IL-17A mRNA; Open bars, Foxp3 mRNA.

If the size of the different subpopulations is taken into account, it is apparent that CD8^+^IELs are dominating and responsible for 69±13% of all the IL-17A mRNA expressed in the three IEL subsets together (*P*<0.01 and <0.001 compared to γδ^+^IELs and CD4^+^IELs, respectively; Figure 4).

In contrast to IL-17A mRNA, Foxp3 mRNA showed the highest expression levels in CD4^+^IELs (Figure 3b) also in Untreated CD and this subset was responsible for most of the Foxp3 mRNA (81±19%, *P*<0.05 and <0.01 compared to γδ^+^IELs and CD8^+^IELs, respectively; Figure 4). In Controls, the CD4^+^IELs were responsible for 70–80% of the low levels of both IL-17A and Foxp3 mRNA (data not shown).

### CD Associated Bacteria Cause an IL-17A Response Upon *ex vivo* Challenge of Biopsies from Treated CD Patients and Influence the IL-17A Response to Gluten Digest

**Figure 5:**
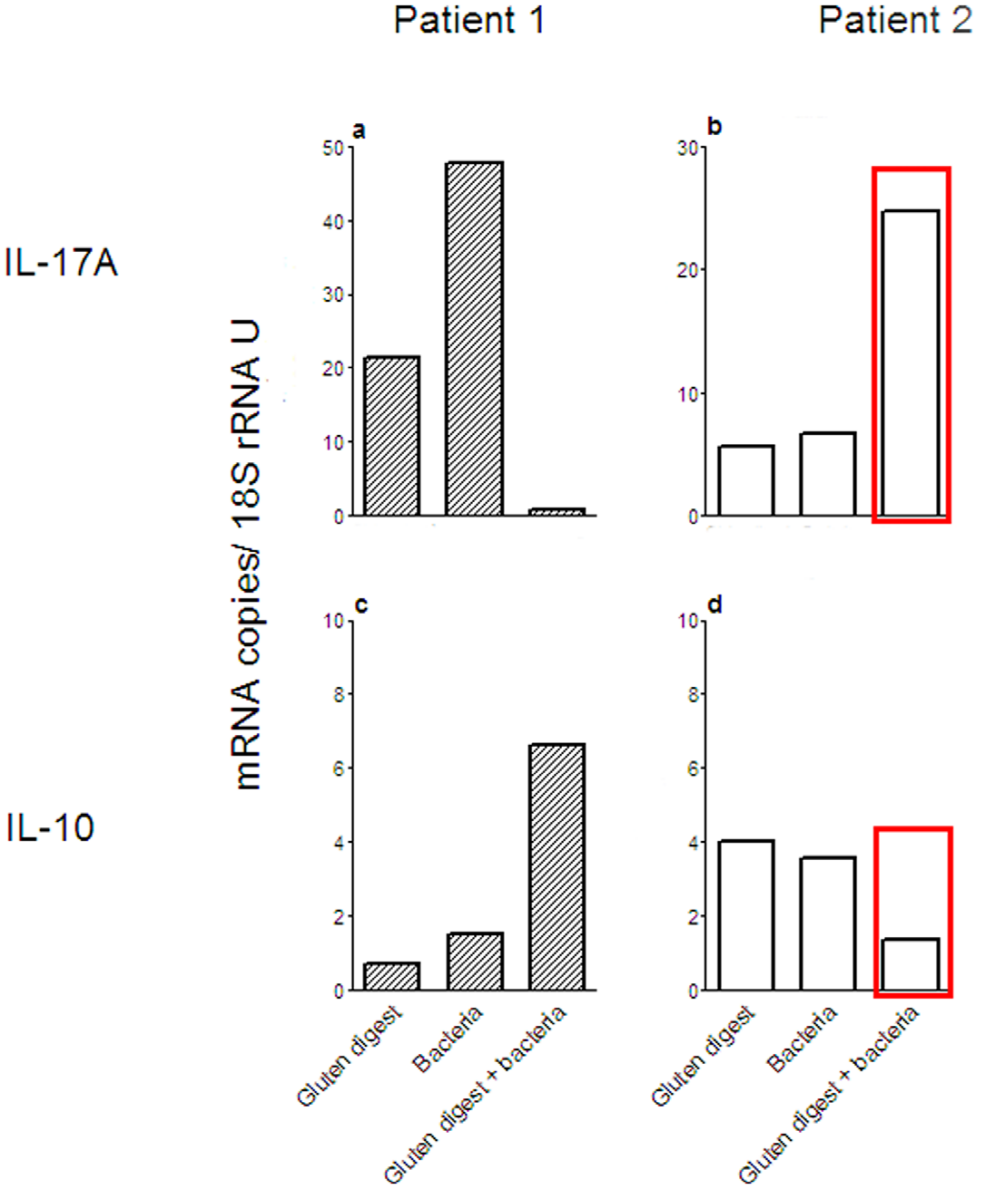
The IL-17A responses of ex vivo challenged biopsies show two different patterns. Expression levels of IL-17A (**a**–**b**) and IL-10 (**c**–**d**) mRNAs were determined in biopsies from treated CD patients after challenge *ex vivo* with gluten digest (Gluten digest), a mixture of CD associated bacteria (Bacteria), a combination of gluten digest and the mixture of CD associated bacteria (Gluten digest+bacteria), or incubated with medium alone. Bars indicate the mRNA expression level for the indicated stimulant of each biopsy after subtraction of the mRNA level in the medium control for Patient #1, born 1996 (**a**, **c**), and Patient #2, born 2006 (**b**, **d**). Determinations were done in triplicates. Red boxes in (**b**) and (**d**) indicate the responses to challenge with the combination of gluten digest and a mixture of CD associated bacteria in the whole biopsy before fractionation into T cell subsets for which the results are shown in **Figure** 7.

The expression levels of the cytokine mRNAs and Foxp3 mRNA were next determined in *ex vivo* challenged biopsies from patients with inactive disease (Treated CD). That the patients included in these experiments had inactive disease was verified by determining the cytokine mRNA levels in a small piece of the freshly taken biopsies. The levels were comparable to those of the Treated CD patient group presented in **Figure** 1. The biopsies were challenged with either a gluten digest, a mixture of *Prevotellas*, *L. umeaense* and *A. graevenitzii* bacteria isolated from proximal jejunum of CD patients, or a combination of gluten digest and the mixture of CD associated bacteria. Challenge with gluten digest caused IL-17A and/or IL-10 up-regulation in all cases but one (**Table** 3). Up-regulation of IFN-γ was seen in only a few cases (2/8; **Table** 3). The latter two cases also had elevated levels of IL-17A and IL-10 mRNAs. One sample responded with up-regulation of Foxp3 mRNA only. Notably, challenge with CD associated bacteria alone caused up-regulation of IL-10 in the majority of cases and IL-17A in half of them, while the response to the combination of gluten digest and bacteria caused up-regulation of IL-17A and IL-10 in 6/8 and 4/8 cases, respectively (**Table** 3). The response to the combination of gluten digest plus CD associated bacteria showed two different patterns, either there was a suppressed IL-17A response and enhanced IL-10 response (Patient #1; **Figure** 5a, c) or there was an enhanced expression of IL-17A mRNA and decreased IL-10 level (Patient #2, **Figure** 5b, d).

**Table 3:** *Ex vivo* challenge of small intestinal biopsies of treated CD patients with gluten digest and CD associated bacteria caused increased expression of IL-17A and IL-10 mRNAs.

	Frequencies of biopsies responding with increased mRNA level of:			
Stimulant:	IL-17A	IFN-γ	IL-10	Foxp3
**Gluten digest**	6/8^1^	2/8	6/8	3/8
**CD associated bacteria**	4/8	3/8	6/8	2/8
**Gluten digest plus CD associated bacteria**	6/8	3/8	4/8	1/8

1 Number of biopsies giving an mRNA level above the sham-treated medium control upon challenge with the indicated stimulant over the total number of biopsies subjected to the indicated challenge.

**Figure 6:**
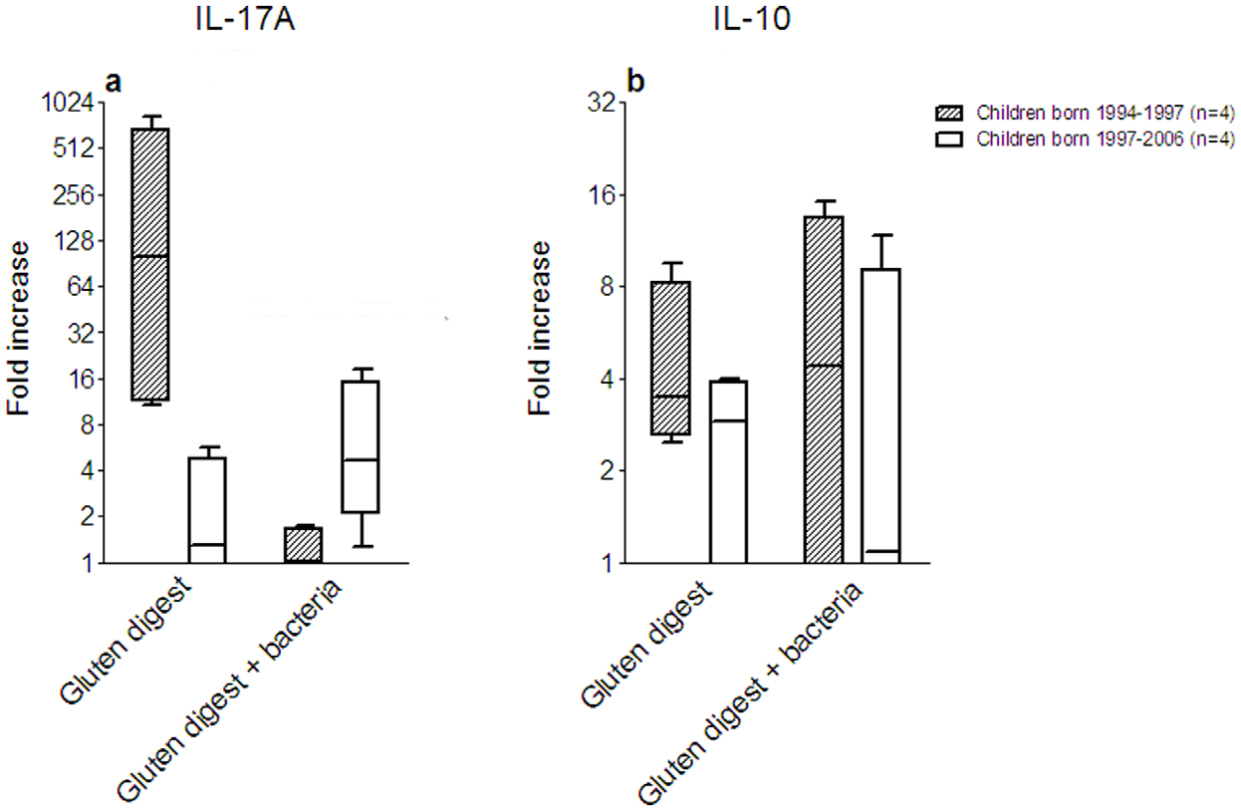
CD associated bacteria suppress the IL-17A response to gluten in children born during the “epidemic”. IL-17A (**a**) and IL-10 (**b**) responses to *ex vivo* challenge with gluten digest alone (Gluten digest) and the combination of gluten digest and a mixture of CD associated bacteria (Gluten digest+bacteria) shown as fold increase calculated as the ratio over the individual medium control for patients #1 through 8 and grouped according to the IL-17A response to challenge with gluten digest alone. Horizontal bars indicate medians, boxes indicate the 25^th^ to 75^th^ IQR, and whiskers indicate the range. Filled boxes and bars: Treated CD patients with strong IL-17A responses to challenge with gluten digest alone, all born during the Swedish CD epidemic. Open boxes and bars: Treated CD patents with weak IL-17A responses to challenge with gluten digest alone, all born after the epidemic. n = 4 in each group.

Based on their IL-17A mRNA response to gluten digest, calculated as the ratio between the level in the challenged biopsy and the medium control, the patients were divided into 2 groups (*P*<0.05; n = 4 per group). This grouping of the patients revealed that the children who responded most strongly to gluten digest alone responded poorly to the combination of gluten digest and the mixture of CD associated bacteria (**Figure** 6a). Interestingly, these patients were born 1994–1997, i.e. during the Swedish CD epidemic. Conversely the children who showed only a weak IL-17A response to challenge with gluten digest alone showed a stronger IL-17A response to the combination of gluten digest and CD associated bacteria (**Figure** 6a). Children in the latter group were born 1997–2006, i.e. after the epidemic. The former group also tended to show a higher IL-10 response to gluten digest alone than the latter group (**Figure** 6b). The children who showed a strong IL-17A response to challenge with gluten digest alone also tended to show a stronger IL-10 response. However, there was no difference between the groups in their IL-10 response to the combination of gluten digest and CD associated bacteria (**Figure** 6b).

**Figure 7:**
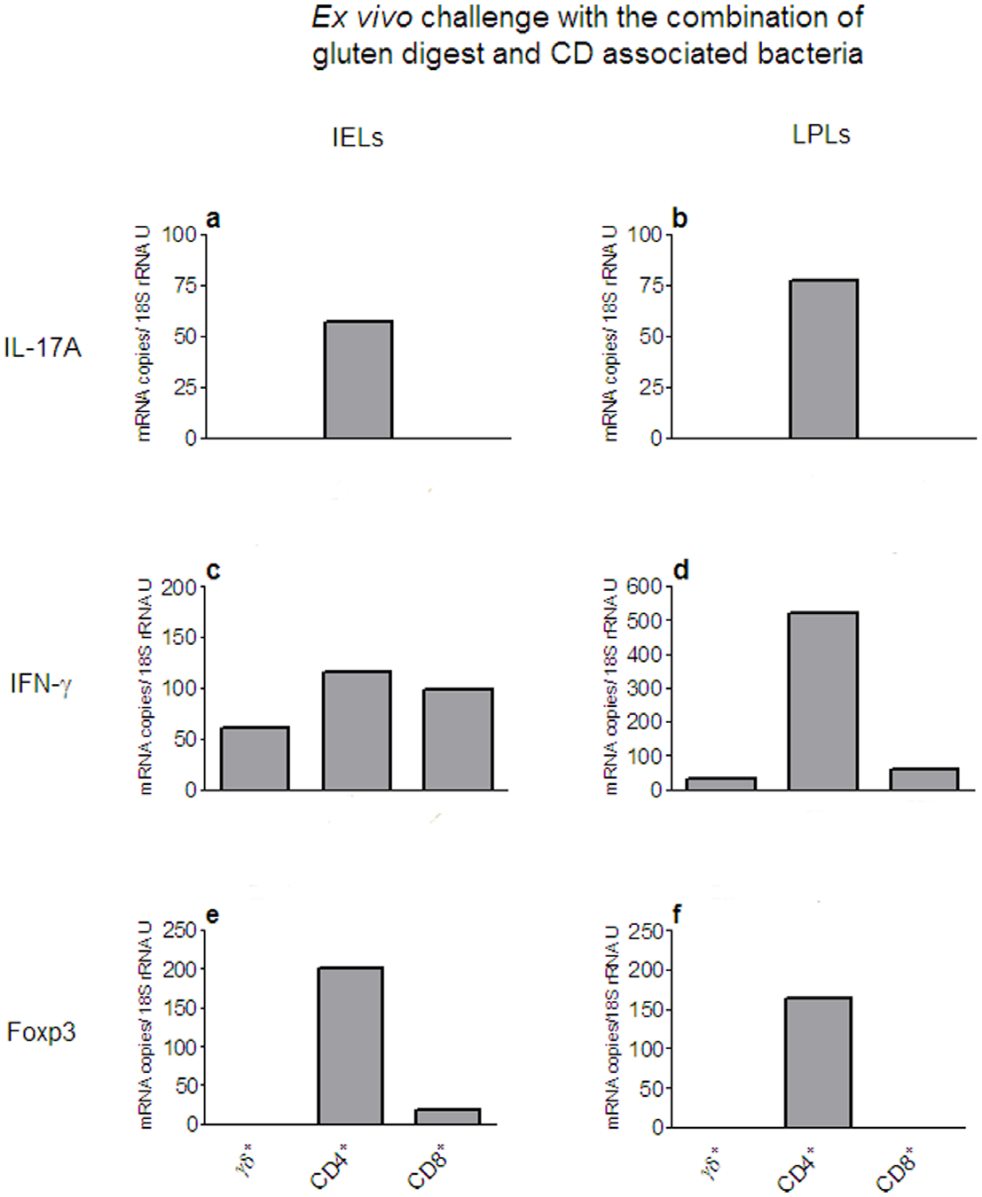
Ex vivo challenge with gluten digest combined with CD associated bacteria provokes a Th17 response. Expression levels of IL-17A **(a**, **b**), IFN-γ (**c**, **d**), and Foxp3 (**e**, **f**) mRNAs determined in TCR-γδ^+^ (γδ^+^)-, CD4^+^ TCR-γδ^−^ (CD4^+^)-, and CD8^+^ CD4^−^ TCR-γδ^−^ (CD8^+^)-cells retrieved from IELs and LPLs isolated from an intestinal biopsy of patient #2 in **Figure** 5 after challenge *ex vivo* with a combination of gluten digest and a mixture of CD associated bacteria. The IL-17A and IL-10 responses in the whole biopsy are indicated by red boxes in **Figure** 5 (**b**) and (**d**). Bars indicate the mRNA expression level of each T cell subtype. Determinations were done in triplicates.

In two patients we had the possibility to analyse the response of different T cell subsets in biopsies challenged *ex vivo* with the combination of gluten digest and the mixture of CD associated bacteria. Both were born after the epidemic and gave similar results. **Figure** 7 shows the results for one of the patients. From this patient a sample of the intact, challenged biopsy was also analysed (patient #2 marked with red box in **Figure** 5b and d). TCR-γδ^+^-, CD4^+^-, and CD8^+^ cells were retrieved from IELs and LPLs isolated from the challenged biopsies and the expression levels of IL-17A, IL-10, IFN-γ and Foxp3 mRNAs were determined subsequently in the T cell subsets. Interestingly, CD4^+^ cells showed the highest IL-17A mRNA expression levels, both of the IELs and the LPLs (**Figure** 7a, b). IL-10 mRNA was not detected in any of the T cell subpopulations (data not shown), which suggests that the low level in the intact biopsy of this individual might come from non-lymphoid immune cells (marked with red box in **Figure** 5d). All three IEL subsets had similar expression levels of IFN-γ mRNA while the IFN-γ response in LP was mainly by the CD4^+^ cells (**Figure** 7c, d). As expected the expression levels of Foxp3 mRNA were highest in CD4^+^ cells (**Figure** 7e, f).

## Discussion

This study shows that IL-17A production is a characteristic feature of active CD. At diagnosis children with CD had highly elevated levels of IL-17A mRNA in their jejunal mucosa that returned to background levels on a gluten-free diet. Moreover, IL-17A production was at least partly due to a reaction against gluten because *ex vivo* challenge of biopsies of treated symptom-free CD patients with gluten peptides increased IL-17A mRNA levels. These findings confirm the previous studies suggesting a role for IL-17A in CD, i.e. demonstrating IL-17A expression in active CD, IL-17A responses to *ex vivo* challenge with gliadin peptides and gliadin specific Th17 cells in CD patients [19], [20], [21], [31]. We found that *ex vivo* challenge of intestinal biopsies from CD patients with gluten peptides caused increased IL-17A levels in most samples while only a few responded with concomitant increase in the IFN-γ level. One explanation for this somewhat unexpected observation could be that the kinetics for induction of IL-17A is faster than for IFN-γ.

In the present study we take the analysis of active, untreated CD further by performing immunohistochemical analysis combined with morphometry of the small intestinal mucosa for cells expressing the IL-17A protein. A significantly increased frequency of IL-17A^+^ cells, particularly in the epithelium, was found in active CD compared to controls. These results are in line with a recent study by Lahdenperä et al. [32] in which IL-17A^+^ cells were demonstrated in the small intestinal LP with an increased average frequency in active CD. In their study, however, the epithelium was not analyzed.

**Figure 8:**
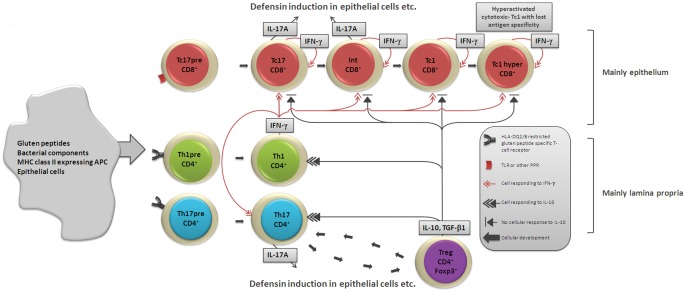
Hypothetical scenario of the immune situation in the small intestinal mucosa during the inflammatory reaction. The reaction to challenge at the mucosal lining with gluten peptides and components of CD associated bacteria induces at process during which the intraepithelial Tc17 cells (red) become activated and gradually transform to hyperactivated cytotoxic T lymphocytes with lost antigen specificity. IFN-γ drives the development both in an autocrine manner with Tc17 cells converting to Tc1 cells and paracrine manner through Th1 cells (green) specifically stimulated by gluten peptides presented on HLA-DQ2/DQ8. Parallel activation of Tregs (violet) occurs. These Tregs secrete IL-10 that down-regulates Th17 (blue) and Th1 cells but the Tc17 cells are resistant to down-regulation. IL-17A secreted by Tc17 and Th17 cells upon activation by gluten peptides and/or bacterial components promotes both inflammation and an antibacterial response including for example defensin production by epithelial cells.

New findings are that both Th17 and Tc17 cells contribute significantly to the IL-17A response in the inflamed small intestinal mucosa of patients with active CD and that IL-17A mRNA levels in the mucosa correlate with the mRNA levels of IFN-γ and Foxp3 mRNAs but not with the level of IL-10 mRNA. Tc17 cells, which are CD8^+^ cells, are mainly present in the epithelium and we show that they are responsible for most of the IL-17A activity in this compartment. In active CD, IL-17A producing IELs, i.e. the Tc17 cells, were more than 6 times as abundant as in IELs of controls and was ≈10 times higher than in LPLs in patients with untreated CD. Furthermore, only a 1.5 fold increase of IL-17A producing LPLs was seen in active CD compared to controls. IL-17A produced by the Tc17 cells in the epithelium may contribute significantly to the antimicrobial defense by recruitment of immune cells to infected sites and by up-regulation of anti-microbial peptides [33]–[35]. That CD has traits of an antibacterial reaction is in concert with our previous finding that levels of the α-defensins, HD-5 and HD-6, as well as lysozyme are increased in the small intestinal mucosa in active CD [14]. Generally, Tc17 cells are found in the intestinal mucosa and in the lung and shown to have functional plasticity, low cytolytic function, involvement in the immune response to viruses and in the disease process of certain autoimmune diseases like psoriasis [36]–[41]. In fact, Tc17 cells are expanded in psoriasis skin lesions [37]. We demonstrate that CD8^+^IELs in the inflamed mucosa in active CD produce both IL-17A and IFN-γ (this study and [9]). Taken together with the findings that Tc17 cells can show functional plasticity, simultaneously produce IL-17A and IFN-γ and convert into Tc1 cells [40], [41], we hypothesize, as depicted in **Figure** 8, that the Tc17 cells undergo functional plasticity during the disease process in untreated CD developing into the characteristic CD8^+^ IELs producing large amounts of IFN-γ and finally develop into the hyperstimulated cytotoxic T lymphocytes with lost specificity control as shown by Meresse et al. [42]. Our results furthermore suggest a parallel activation of Th17/Tc17 cells and Tregs. Mouse studies have shown that pro-inflammatory autoimmune Th17 cells are controlled in the small intestine partly by conversion to Tregs [43], [44]. In CD this control seems to be insufficient and it is even possible that Tregs convert into Th17 cells [44], [45]. One explanation for the fact that high levels of IL-17A occur in the presence of elevated levels of down-regulatory cytokines like IL-10 in active CD might be that the Tc17 cells are resistant to inhibition by Tregs (**Figure** 8), as was shown to be the case for Tc17 cells in psoriatic lesions [37].

The CD associated bacteria investigated in this study influenced the IL-17A expression in the small intestinal mucosa of CD patients in two ways. Firstly they were capable of inducing an IL-17A response on their own suggesting that the increased IL-17A response seen in active CD in part could be directed against the CD associated bacteria and secondly, addition of these bacteria during *ex vivo* challenge with gluten digest influenced the IL-17A response, either suppressing or enhancing it depending on whether the patient had a strong response to gluten digest alone or not. Therefore we believe that the CD associated bacteria play an important role in determining the magnitude of the IL-17A response. An IL-17A response to the CD associated bacteria might contribute to break the tolerance to gluten with the consequence that the individual develops CD. We used a mixture of CD associated bacteria for challenge of the biopsies. The mixture included *L. umeaense*, a spore-forming, segmented filamentous bacterium that seems to adhere to small intestinal epithelium and hence shows great resemblance to the SFB (segmented, filamentous bacteria belonging to the genus *Candidatus*) that proved of importance for development of the gut immune system in mice and in particular the development of mucosal Th17 cells [46]–[48]. Recent studies have shown that *L. umeaense* and SFB are different species but are indeed related and both belong to the *Lachnospiraceae* family [16], [49]. It is possible that more than one species of adherent bacteria is capable of driving development of Th17 cells or that *L. umeaense* is the counterpart in humans to SFB in mice. An additional, not mutually exclusive, possibility is that *L. umeaense* bacteria stimulate Tregs to IL-10 production and/or conversion of Th17 cells to Tregs. It has been shown that a mixture of 46 indigenous intestinal bacterial strains of the genus *Clostridium* induces Tregs in mouse colon [50]. Several of the bacterial strains belonged to the XIVa cluster of *Clostridium* [51] that is closely related to the new genus *Lachoanaerobaculum* [16].

When the mixture of the CD associated bacteria was added during challenge with gluten peptides two different patterns in IL-17A response emerged compared to the response to challenge with gluten peptides alone, i.e. enhancement or suppression. Interestingly it turned out that those patients who showed a suppressed IL-17A response when CD associated bacteria were included were all children born during the Swedish CD epidemic. In contrast, children born after the epidemic all showed an enhancement of the IL-17A response. Our previous study on the microbiota in the small intestine showed that adherent rod-shaped bacteria detectable by scanning electron microscopy were most frequent in young CD patients during the epidemic [14], [15]. The fact that it 10 years later was possible to isolate CD associated rod-shaped bacteria from biopsies of patients born during the epidemic suggests that the patients acquired an unfavorable composition of their small intestinal microbiota at that time and that dysbiosis of the microbiota has prevailed since then [15]. Thus, it is possible that the different outcomes of adding CD associated bacteria are dependent on differences in the resident microbiota of the patients. The suppressed IL-17A response in patients born during the epidemic could mean that they have developed Tregs directed against CD associated bacteria present in their resident microbiota or that they have become impaired in their capacity to generate an adequate anti-bacterial response. Notably, the response to challenge with gluten peptides alone was significantly higher in these children than in those born after the epidemic suggesting that they are strongly gluten reactive and that the level of the anti-gluten response is enhanced by the components in the resident microbiota. Other groups have also suggested that intestinal dysbiosis may be a contributing factor for CD [52]. In accordance with this notion, two recent studies report differences between the fecal microbiota of infants genetically predisposed for developing CD and those who are not [53], [54]. A link between dysbiosis of human gut microbiota with outgrowth of potential pathogenic bacteria and other immune disorders has previously been suggested. Of particular interest in relation to CD are the reports showing that patients with inflammatory bowel disease have a composition of their gut microbiota that differs from healthy individuals [46].

The enhanced IL-17A response seen in biopsies from CD patients born after the epidemic upon challenge with a combination of gluten peptides and a mixture of CD associated bacteria differed from the IL-17A response seen in fresh biopsies from CD patients with active disease. Thus, CD4^+^ cells, Th17 cells, totally dominated as the cellular source of IL-17A in the challenged biopsies, both within the epithelium and in the LP. This could be due to slower kinetics for raising a Tc17 cell response than for a Th17 cell response in the epithelium or that an epithelial reaction dominated by Tc17 cells is a feature of the immune situation in the mucosa of patients presenting with the disease in whom the Tc17 cells are converted into IFN-γ producing Tc1 cells as the disease progresses (**Figure** 8).

A limitation of our study is that *ex vivo* challenge experiments require fresh biopsies from symptom-free CD patients on a gluten-free diet. Biopsies included in this study were all collected based on clinical requirements for the diagnostic procedure [55]. Collection of a second biopsy after treatment with gluten-free diet is therefore performed only in a fraction of the patients and hence only few biopsies from treated CD patients are available for research and consequently the number of biopsies subjected to *ex vivo* challenge in this study is limited.

Collectively, the data presented here suggest that the IL-17A producing cells play a major role in the pathogenesis of CD, that both gluten and CD associated bacteria provoke an IL-17A response in the intestinal mucosa of CD patients and that the magnitude of the adverse IL-17A reaction to gluten is markedly influenced by the composition of the resident microbiota and the amount of CD associated bacteria present. The results are in line with the hypothesis that circumstances causing disturbances when the resident microbiota of the small intestine is established can lead to long-lasting dysbiosis with increased amounts of one or several of the CD associated bacterial species we have identified. These changes in microbiota may influence the magnitudes of IL-17A responses to gluten and be risk factors for contraction of CD in predisposed children. Possibly some of the contradictive results on IL-17A responses in CD in the literature [19]–[23], [31], [32] can be explained by the degree of dysbiosis in the individual’s gut microbiota and the presence or absence of the CD associated bacteria.

## References

[1] Sollid LM (2002) Coeliac disease: dissecting a complex inflammatory disorder. Nat Rev Immunol 2: 647–655. 12209133 10.1038/nri885

[2] Tack GJ, Verbeek WH, Schreurs MW, Mulder CJ (2010) The spectrum of celiac disease: epidemiology, clinical aspects and treatment. Nat Rev Gastroenterol Hepatol 7: 204–213. 20212505 10.1038/nrgastro.2010.23

[3] Di Sabatino A, Corazza GR (2009) Coeliac disease. Lancet 373: 1480–1493. 19394538 10.1016/S0140-6736(09)60254-3

[4] Forsberg G, Hernell O, Melgar S, Israelsson A, Hammarström S, et al (2002) Paradoxical coexpression of proinflammatory and down-regulatory cytokines in intestinal T cells in childhood celiac disease. Gastroenterology 123: 667–678. 12198691 10.1053/gast.2002.35355

[5] Sollid LM, Khosla C (2011) Novel therapies for coeliac disease. J Intern Med 269: 604–613. 21401739 10.1111/j.1365-2796.2011.02376.x PMC3101315

[6] Garrote JA, Gómez-Gonzaléz E, Bernardo D, Arranz E, Chirdo F (2008) Celiac disease pathogenesis: The proinflammatory cytokine network. J Pediatr Gastroenterol Nutr 47: 27–32. 10.1097/MPG.0b013e3181818fb9 18667914

[7] Lundin KE, Scott H, Hansen T, Paulsen G, Halstensen TS, et al (1993) Gliadin-specific, HLA-DQ (α1*0501, β1*0201) restricted T cells isolated from the small intestinal mucosa of celiac disease patients. J Exp Med 178: 187–196. 8315377 10.1084/jem.178.1.187 PMC2191064

[8] Gianfrani C, Troncone R, Mugione P, Cosentini E, De Pascale M, et al (2003) Celiac disease association with CD8^+^ T cell responses: identification of a novel gliadin-derived HLA-A2-restricted epitope. J Immunol 170: 2719–2726. 12594302 10.4049/jimmunol.170.5.2719

[9] Forsberg G, Hernell O, Hammarström S, Hammarström M-L (2007) Concomitant increase of IL-10 and pro-inflammatory cytokines in intraepithelial lymphocyte subsets in celiac disease. Int Immunol 19: 993–1001. 17660501 10.1093/intimm/dxm077

[10] Bas A, Forsberg G, Sjöberg V, Hammarström S, Hernell O, et al (2009) Aberrant extrathymic T cell receptor gene rearrangement in the small intestinal mucosa: a risk factor for coeliac disease? Gut 58: 189–195. 18299319 10.1136/gut.2007.125526 PMC2613440

[11] Ivarsson A, Persson LÅ, Nyström L, Ascher H, Cavell B, et al (2000) Epidemic of coeliac disease in Swedish children. Acta Paediatr 89: 165–171. 10709885 10.1080/080352500750028771

[12] Ivarsson A, Hernell O, Stenlund H, Persson LÅ (2002) Breast-feeding protects against celiac disease. Am J Clin Nutr 75: 914–921. 11976167 10.1093/ajcn/75.5.914

[13] Myléus A, Ivarsson A, Webb C, Danielsson L, Hernell O, et al (2009) Celiac disease revealed in 3% of Swedish 12-year-olds born during an epidemic. J Pediatr Gastroenterol Nutr 49: 170–176. 19516192 10.1097/MPG.0b013e31818c52cc

[14] Forsberg G, Fahlgren A, Hörstedt P, Hammarström S, Hernell O, et al (2004) Presence of bacteria and innate immunity of intestinal epithelium in childhood celiac disease. Am J Gastroenterol 99: 894–904. 15128357 10.1111/j.1572-0241.2004.04157.x

[15] Ou G, Hedberg M, Hörstedt P, Baranov V, Forsberg G, et al (2009) Proximal small intestinal microbiota and identification of rod-shaped bacteria associated with childhood celiac disease. Am J Gastroenterol 104: 3058–3067. 19755974 10.1038/ajg.2009.524

[16] Hedberg ME, Moore ER, Svensson-Stadler L, Hörstedt P, Baranov V, et al (2012) *Lachnoanaerobaculum* a new genus in *Lachnospiraceae;* characterization of *Lachnoanaerobaculum umeaense* gen. nov., sp. nov., isolated from human small intestine, *Lachnoanaerobaculum orale* gen. nov., sp. nov., isolated from saliva and reclassification of *Eubacterium saburreum* (Prévot) Holdeman and Moore 1970 as *Lachnoanaerobaculum saburreum* comb. nov. 2011. Int J Syst Evol Microbiol 62: 2685–2690. 22228654 10.1099/ijs.0.033613-0 PMC3541798

[17] Miossec P, Korn T, Kuchroo VK (2009) Interleukin-17 and type 17 helper T cells. N Engl J Med 361: 888–898. 19710487 10.1056/NEJMra0707449

[18] Costa VS, Mattana TCC, Rossi da Silva ME (2010) Unregulated IL-23/IL-17 immune response in autoimmune diseases. Diabets Res Clin Practice 88: 222–226. 10.1016/j.diabres.2010.03.014 20392505

[19] Castellanos-Rubio A, Santin I, Irastorza I, Castaño L, Vitoria JC, et al (2009) Th17 (and Th1) signatures of intestinal biopsies of CD patients in response to gliadin. Autoimmunity 42: 69–73. 19127457 10.1080/08916930802350789

[20] Monteleone I, Sarra M, Del Vecchio Blanco G, Paoluzi OA, Franzè E, et al (2010) Characterization of IL-17A-producing cells in celiac disease mucosa. J Immunol 184: 2211–2218. 20061410 10.4049/jimmunol.0901919

[21] Fernández S, Molina IJ, Romero P, González R, Peña J, et al (2011) Characterization of gliadin-specific Th17 cells from the mucosa of celiac disease patients. Am J Gastroenterol 106: 528–538. 21206487 10.1038/ajg.2010.465

[22] Bodd M, Ráki M, Tollefsen S, Fallang LE, Bergseng E, et al (2010) HLA-DQ2-restricted gluten-reactive T cells produce IL-21 but not IL-17 or IL-22. Mucosal Immunol 6: 594–601. 10.1038/mi.2010.36 20571486

[23] La Scaleia R, Barba M, Di Nardo G, Bonamico M, Oliva S, et al (2012) Size and dynamics of mucosal and peripheral IL-17A+ T-cell pools in pediatric age, and their disturbance in celiac disease. Mucosal Immunol 5: 513–523. 22569303 10.1038/mi.2012.26

[24] Marsh MN (1990) Grains of truth: evolutionary changes in small intestinal mucosa in response to environmental antigen challenge. Gut 31: 111–114. 2180789 10.1136/gut.31.1.111 PMC1378351

[25] Sjöström H, Lundin KE, Molberg O, Körner R, McAdam SN, et al (1998) Identification of a gliadin T-cell epitope in celiac disease: General importance of gliadin deamidation for intestinal T-cell recognition. Scand J Immunol 48: 111–115. 9716100 10.1046/j.1365-3083.1998.00397.x

[26] Bas A, Hammarström S, Hammarström M-L (2003) Extrathymic TCR gene rearrangement in human small intestine: Identification of new splice forms of recombination activating gene-1 mRNA with selective tissue expression. J Immunol 171: 3359–3371. 14500629 10.4049/jimmunol.171.7.3359

[27] Lundqvist C, Baranov V, Teglund S, Hammarström S, Hammarström M-L (1994) Cytokine profile and ultrastructure of intraepithelial γδ T cells in chronically inflamed human gingiva suggest a cytotoxic effector function. J Immunol 153: 2302–2312. 8051426

[28] Morgan ME, van Bilsen JH, Bakker AM, Heemskerk B, Schilham MW, et al (2005) Expression of FOXP3 mRNA is not confined to CD4^+^CD25^+^ T regulatory cells in humans. Hum Immunol 66: 13–20. 15620457 10.1016/j.humimm.2004.05.016

[29] West CE, Hernell O, Andersson Y, Sjöstedt M, Hammarström M-L (2012) Probiotic effects on T-cell maturation in infants during weaning. Clin Exp Allergy 42: 540–549. 22417212 10.1111/j.1365-2222.2011.03941.x

[30] Bas A, Forsberg G, Hammarström S, Hammarström M-L (2004) Utility of the housekeeping genes 18S rRNA, β-actin and glyceraldehyde-3-phosphate-dehydrogenase for normalization in real-time quantitative reverse transcriptase-polymerase chain reaction analysis of gene expression in human T lymphocytes. Scand J Immunol 59: 566–573. 15182252 10.1111/j.0300-9475.2004.01440.x

[31] Di Sabatino A, Rovedatti L, Vetrano S, Vidali F, Biancheri P, et al (2011) Involvement of CD40-CD40 ligand in uncomplicated and refractory celiac disease. Am J Gastroenterol 106: 519–520. 21139574 10.1038/ajg.2010.450

[32] Lahdenperä AI, Hölttä V, Rouhtula T, Salo HM, Orivuori L, et al (2012) Up-regulation of small intestinal interleikin-17 immunity in untreated coeliac disease but not in potential coeliac disease or in type 1 diabetes. Clin Exp Immunol 167: 226–234. 22235998 10.1111/j.1365-2249.2011.04510.x PMC3278688

[33] Kao C-Y, Chen Y, Thai P, Wachi S, Huang F, et al.. (2004) IL-17 markedly up-regulates β-defensin-2 expression in human airway epithelium via JAK and NF-κB signaling pathways. J Immunol 173, 3482–3491. 10.4049/jimmunol.173.5.3482 15322213

[34] Liang SC, Tan XY, Luxenberg DP, Karim R, Dunussi-Joannopoulos K, et al (2006) Interleukin (IL)-22 and IL-17 are coexpressed by Th17 cells and cooperatively enhance expression of antimicrobial peptides. J Exp Med 203: 2271–2279. 16982811 10.1084/jem.20061308 PMC2118116

[35] Rahman A, Fahlgren A, Sundstedt C, Hammarström S, Danielsson Å, et al (2011) Chronic colitis induces expression of β-defensins in murine intestinal epithelial cells. Clin Exp Immunol 163: 123–130. 21039426 10.1111/j.1365-2249.2010.04282.x PMC3010919

[36] Ciric B, El-behi M, Cabrera R, Zhang G-X, Rostami A (2009) IL-23 drives pathogenic IL-17-producing CD8^+^ T cells. J Immunol 182: 5296–5305. 19380776 10.4049/jimmunol.0900036

[37] Ortega C, Fernández-A S, Carrillo JM, Romero P, Molina IJ, et al (2009) IL-17-producing CD8^+^ T lymphocytes from psoriasis skin plaques are cytotoxic effector cells that secrete Th17-related cytokines. J Leukoc Biol 86: 435–443. 19487306 10.1189/JLB.0109046

[38] Huber M, Heink S, Grothe H, Guralnik A, Reinhard K, et al (2009) A Th17-like developmental process leads to CD8^+^ Tc17 cells with reduced cytotoxic activity. Eur J Immunol 39: 1716–1725. 19544308 10.1002/eji.200939412

[39] Hamada H, Garcia-Hernandez Mde L, Reome JB, Misra SK, Strutt TM, et al (2009) Tc17, a unique subset of CD8 T cells that can protect against lethal influenza challenge. J Immunol 182: 3469–3481. 19265125 10.4049/jimmunol.0801814 PMC2667713

[40] Yen H-R, Harris TJ, Wada S, Grosso JF, Getnet D, et al (2009) Tc17 CD8 T cells: functional plasticity and subset diversity. J Immunol 183: 7161–7168. 19917680 10.4049/jimmunol.0900368 PMC3082359

[41] Yeh N, Glosson NL, Wang N, Guindon L, McKinley C, et al (2010) Tc17 cells are capable of mediating immunity to vaccinia virus by acquisition of a cytotoxic phenotype. J Immunol 185: 2089–2098. 20624947 10.4049/jimmunol.1000818 PMC2916954

[42] Meresse B, Curran SA, Ciszewski C, Orbelyan G, Setty M, et al (2006) Reprogramming of CTLs into natural killer-like cells in celiac disease. J Exp Med 203: 1343–1355. 16682498 10.1084/jem.20060028 PMC2121214

[43] Esplugues E, Huber S, Gagliani N, Hauser AE, Town T, et al (2011) Control of T_H_17 cells occurs in the small intestine. Nature 475: 514–518. 21765430 10.1038/nature10228 PMC3148838

[44] Bilate AM, Lafaille JJ (2012) Induced CD4^+^Foxp3^+^ regulatory T cells in immune tolerance. Annu Rev Immunol 30: 733–758. 22224762 10.1146/annurev-immunol-020711-075043

[45] Ayyoub M, Deknuydt F, Raimbaud I, Dousset C, Leveque L, et al (2009) Human memory FOXP3^+^ Tregs secrete IL-17 ex vivo and constitutively express the T_H_17 lineage-specific transcription factor RORγt. Proc Natl Acad Sci U S A 106: 8635–8640. 19439651 10.1073/pnas.0900621106 PMC2688993

[46] Honda K, Littman DR (2012) The microbiome in infectious disease and inflammation. Annu Rev Immunol 30: 759–795. 22224764 10.1146/annurev-immunol-020711-074937 PMC4426968

[47] Gaboriau-Routhiau V, Rakotobe S, Lécuyer E, Mulder I, Lan A, et al (2009) The key role of segmented filamentous bacteria in the coordinated maturation of gut helper T cell responses. Immunity 31: 677–689. 19833089 10.1016/j.immuni.2009.08.020

[48] Ivanov II, Atarashi K, Manel N, Brodie EL, Shima T, et al (2009) Induction of intestinal Th17 cells by segmented filamentous bacteria. Cell 139: 485–498. 19836068 10.1016/j.cell.2009.09.033 PMC2796826

[49] Pamp SJ, Harrington ED, Quake SR, Relman DA, Blainey PC (2012) Single-cell sequencing provides clues about the host interactions of segmented filamentous bacteria (SFB). Genome Res 22: 1107–1119. 22434425 10.1101/gr.131482.111 PMC3371716

[50] Atarashi K, Tanoue T, Shima T, Imaoka A, Kuwahara T, et al (2011) Induction of colonic regulatory T cells by indigenous Clostridium species. Science 331: 337–341. 21205640 10.1126/science.1198469 PMC3969237

[51] Collins MD, Lawson PA, Willems A, Cordoba JJ, Fernandez-Garayzabal J, et al (1994) The phylogeny of the genus *Clostridium*: proposal of five new genera and eleven new species combinations. Int J Syst Bacteriol 44: 812–826. 7981107 10.1099/00207713-44-4-812

[52] Sanz Y, De Palma G, Laparra M (2011) Unraveling the ties between celiac disease and intestinal microbiota. Int Rev Immunol 30: 207–218. 21787226 10.3109/08830185.2011.599084

[53] Sellitto M, Bai G, Serena G, Fricke WF, Sturgeon C, et al (2012) Proof of concept of microbiome-metabolome analysis and delayed gluten exposure on celiac disease autoimmunity in genetically at-risk infants. PLoS ONE 7: e33387. 22432018 10.1371/journal.pone.0033387 PMC3303818

[54] De Palma G, Capilla A, Nova E, Castillejo G, Varea V, et al (2012) Influence of milk-feeding type and genetic risk of developing coeliac disease on intestinal microbiota of infants: The PROFICEL study. PLoS ONE 7: e30791. 22319588 10.1371/journal.pone.0030791 PMC3272021

[55] Husby S, Koletzko S, Korponay-Szabó IR, Mearin ML, Phillips A, et al (2012) ESPGHAN Working Group on Coeliac Disease Diagnosis, ESPGHAN Gastroenterology Committee, European Society for Pediatric Gastroenterology, Hepatology, and Nutrition; European Society for Pediatric Gastroenterology, Hepatology, and Nutrition guidelines for the diagnosis of coeliac disease. J Pediatr Gastroenterol Nutr 54: 136–160. 22197856 10.1097/MPG.0b013e31821a23d0

